# The Leukemia-Specific Fusion Gene *ETV6/RUNX1* Perturbs Distinct Key Biological Functions Primarily by Gene Repression

**DOI:** 10.1371/journal.pone.0026348

**Published:** 2011-10-20

**Authors:** Gerhard Fuka, Maximilian Kauer, Reinhard Kofler, Oskar A. Haas, Renate Panzer-Grümayer

**Affiliations:** 1 Children's Cancer Research Institute, St. Anna Kinderkrebsforschung, Vienna, Austria; 2 Division of Molecular Pathophysiology, Tyrolean Cancer Research Institute and Biocenter, Innsbruck Medical University, Innsbruck, Austria; 3 St. Anna Kinderspital, Vienna, Austria; National Institute of Health, United States of America

## Abstract

**Background:**

*ETV6/RUNX1* (*E/R*) (also known as *TEL/AML1*) is the most frequent gene fusion in childhood acute lymphoblastic leukemia (ALL) and also most likely the crucial factor for disease initiation; its role in leukemia propagation and maintenance, however, remains largely elusive. To address this issue we performed a shRNA-mediated knock-down (KD) of the *E/R* fusion gene and investigated the ensuing consequences on genome-wide gene expression patterns and deducible regulatory functions in two *E/R*-positive leukemic cell lines.

**Findings:**

Microarray analyses identified 777 genes whose expression was substantially altered. Although approximately equal proportions were either up- (KD-UP) or down-regulated (KD-DOWN), the effects on biological processes and pathways differed considerably. The E/R KD-UP set was significantly enriched for genes included in the “cell activation”, “immune response”, “apoptosis”, “signal transduction” and “development and differentiation” categories, whereas in the E/R KD-DOWN set only the “PI3K/AKT/mTOR signaling” and “hematopoietic stem cells” categories became evident. Comparable expression signatures obtained from primary *E/R*-positive ALL samples underline the relevance of these pathways and molecular functions. We also validated six differentially expressed genes representing the categories “stem cell properties”, “B-cell differentiation”, “immune response”, “cell adhesion” and “DNA damage” with RT-qPCR.

**Conclusion:**

Our analyses provide the first preliminary evidence that the continuous expression of the *E/R* fusion gene interferes with key regulatory functions that shape the biology of this leukemia subtype. E/R may thus indeed constitute the essential driving force for the propagation and maintenance of the leukemic process irrespective of potential consequences of associated secondary changes. Finally, these findings may also provide a valuable source of potentially attractive therapeutic targets.

## Introduction

The *ETV6/RUNX1* (*E/R*) fusion gene (also known as *TEL/AML1*) is the hallmark of one of the most common genetic subtypes of B-cell precursor acute lymphoblastic leukemia (BCP ALL) in children [1], [2]. The fusion gene encodes a chimeric transcription factor that comprises the N-terminal portion of ETV6 and the almost entire RUNX1 protein and is thought to convert RUNX1 from a transcriptional modulator to a transcriptional repressor of RUNX1 target genes [3]. The current multistep model implies that this gene fusion occurs already during fetal development and constitutes the initiating - although not sufficient - event for neoplastic transformation [4], [5]. The idea that the ensuing gene product might perhaps also be relevant for maintenance of the malignant phenotype is derived from the results of recent experiments, which showed that RNAi-mediated silencing of the endogenous fusion gene reduces *in vitro* cell proliferation and cell survival as well as significantly impairs the *in vivo* repopulation capacity of the treated cells in a xenotransplant mouse model [6] (Fuka et al. manuscript submitted).

Microarray technologies made it possible to define the specific gene expression signatures of specific ALL subgroups, including those with an *E/R* fusion gene [7]–[12]. These diagnostically and clinically relevant molecular patterns derive from the comparison of a differentially expressed set of genes in a given type of leukemia relative to other subgroups included in such analyses. Since particular genetic subgroups can be clearly delineated and distinguished with this approach, it seems likely that primary underlying genetic defects, as for instance *E/R*, are the main determinants of the respective gene expression signature, although the transcriptional derangements will most likely also be modified to a certain extent by other factors, such as secondary genetic alterations. To investigate the specific impact of the chimeric E/R protein on overall gene expression, we knocked down the endogenous fusion gene in two leukemia cell lines utilizing fusion transcript specific short hairpin RNAs (shRNA) and compared the native and suppressed gene expression signatures. We also compared the E/R KD signature with that obtained from primary childhood ALL cases and validated the expression of selected target genes that represented various pathways or cellular functions, which were identified with this approach.

## Results and Discussion

### Defining target genes of E/R knockdown

**Figure 1:**
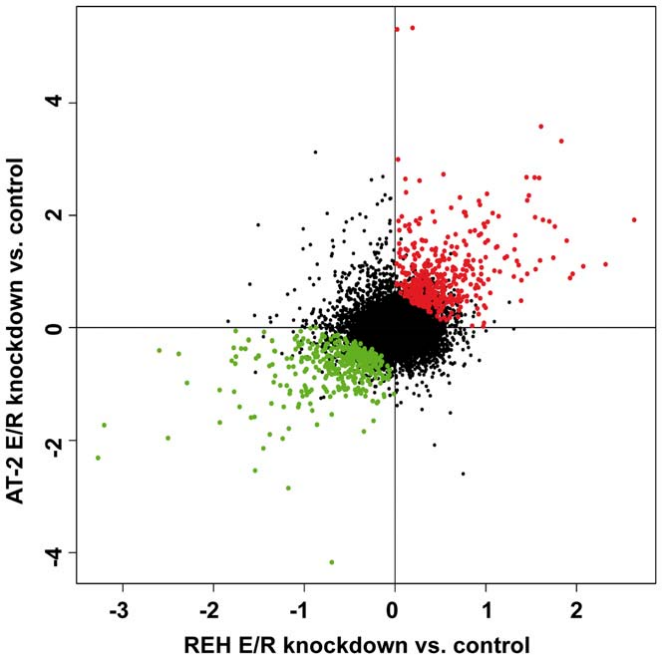
Scatter plot of differential gene expression values upon E/R KD in two cell lines. Each dot represents the mean regulation value (log2-fold change of E/R-repressed versus control cells) of three and two replicas, for REH and AT-2 cell lines, respectively. x-axis: REH cell line, y-axis: AT-2 cell line. Negative values indicate a decrease and positive values an increase in gene expression upon fusion gene KD. Green and red dots depict concordantly and significantly modulated genes in a joint analysis of both cell lines.

We silenced the endogenous fusion protein by lentiviral transduction of shRNA-encoding vectors in the leukemia cell lines REH and AT-2. Detailed information on the experimental design is provided in the Text S1. Expression profiling was performed in cells that were selected for viral integration and stable fusion gene suppression, which resulted in chimeric protein reduction of 50–80% between different experiments (Figure S1). Differentially expressed genes were determined by microarray analyses using three and two biological replicates from independent knock-down (KD) experiments of the REH and AT-2 cell lines, respectively, as well as appropriate control cells that were transduced with a non-targeting shRNA vector. Despite the dissimilar genetic background imposed by different secondary changes in the two cell lines there was a significant correlation of differential gene expression in both models (r = 0.31, *P*<0.0001) (Figure 1). A joint analysis identified 777 genes that were significantly (*P*<0.05) and concordantly up- (KD-UP; n = 403) and down-regulated (KD-DOWN; n = 374) after the knockdown of the E/R fusion gene (Table S1). The top 50 regulated genes are listed in Table 1, along with the log2-fold changes from the array analysis. They include, for instance, the two direct RUNX1 targets *ID2* and *PTPRCAP*. *ID2* encodes a proposed inhibitor of tissue-specific gene expression and *PTPRCAP* is a key regulator of lymphocyte activation (Table S1) [13], [14]. Consistent with the notion that E/R acts as a constitutive repressor of RUNX1 target genes [3], these two genes are repressed in *E/R*-positive leukemias and up-regulated upon fusion gene KD. In contrast to our findings, Wotton et al. report that RUNX1-induced repression of *ID2* is abrogated by E/R. This seemingly controversial result might possibly be explained by a context dependent gene regulation, since Wotton et al. used 3T3 murine fibroblast cells in their experiments. In line with our data, PTPRCAP transcription was found to be repressed by *RUNX1-MTG8* and -*MTG16* fusion genes, two *RUNX1* fusions that are frequently found in acute myeloid leukemia [14]. Furthermore, the regulation of two other genes that are differentially expressed in *E/R*-positive ALL, also concords with our E/R KD results. *CALN1*, a brain-specific member of the calmodulin superfamily, is exclusively over-expressed [10], while *MS4A1* (CD20), a regulator of B-cell activation and proliferation, appears repressed in *E/R*-positive ALL [15].

**Table 1:** Top 50 regulated genes from microarray analysis upon E/R KD.

Entrez Gene ID	Gene name	Gene symbol	E/R KD mean	E/R KD REH	E/R KD AT-2
6689	Spi-B transcription factor	SPIB	2.60	1.61	3.59
931	membrane-spanning 4-domains, subfamily A, member 1	MS4A1	2.58	1.83	3.32
28755	T cell receptor alpha constant	TRAC	2.28	2.64	1.92
197358	NLR family, CARD domain containing 3	NLRC3	2.13	1.59	2.67
4852	neuropeptide Y	NPY	2.11	1.54	2.68
100132169	LOC100132169	LOC100132169	2.06	1.45	2.68
7124	tumor necrosis factor	TNF	1.91	1.47	2.35
6696	secreted phosphoprotein 1	SPP1	1.86	1.46	2.27
28514	delta-like 1	DLL1	1.80	1.70	1.89
7168	tropomyosin 1 (alpha)	TPM1	1.78	1.76	1.80
3759	potassium inwardly-rectifying channel, subfamily J, member 2	KCNJ2	1.77	1.63	1.92
1117	chitinase 3-like 2	CHI3L2	1.76	1.54	1.97
9892	synaptosomal-associated protein, 91 kDa	SNAP91	1.72	2.32	1.13
7940	leukocyte specific transcript 1	LST1	1.72	1.89	1.55
3398	inhibitor of DNA binding 2	ID2	1.70	1.01	2.39
131583	family with sequence similarity 43, member A	FAM43A	1.63	0.53	2.73
140706	chromosome 20 open reading frame 160	C20orf160	1.59	0.92	2.26
971	CD72 molecule	CD72	1.58	2.07	1.09
54541	DNA-damage-inducible transcript 4	DDIT4	1.56	1.14	1.98
4067	v-yes-1 Yamaguchi sarcoma viral related oncogene homolog	LYN	1.56	0.94	2.19
5790	protein tyrosine phosphatase, receptor type, C-associated protein	PTPRCAP	1.56	1.07	2.04
374403	TBC1 domain family, member 10C	TBC1D10C	1.52	0.71	2.32
7490	Wilms tumor 1	WT1	1.50	1.74	1.25
54510	protocadherin 18	PCDH18	1.49	1.32	1.65
8519	interferon induced transmembrane protein 1	IFITM1	1.46	1.96	0.96
6275	S100 calcium binding protein A4	S100A4	1.45	1.02	1.88
9639	Rho guanine nucleotide exchange factor 10	ARHGEF10	1.44	0.27	2.62
4330	meningioma 1	MN1	1.41	0.98	1.85
2014	epithelial membrane protein 3	EMP3	1.41	0.77	2.05
10870	hematopoietic cell signal transducer	HCST	1.41	0.76	2.06
51523	CXXC finger 5	CXXC5	1.41	1.93	0.88
170302	aristaless related homeobox	ARX	−1.42	−2.38	−0.46
3983	actin binding LIM protein 1	ABLIM1	−1.45	−1.77	−1.13
54549	sidekick homolog 2	SDK2	−1.48	−1.17	−1.79
10579	transforming, acidic coiled-coil containing protein 2	TACC2	−1.50	−2.60	−0.40
55107	anoctamin 1	ANO1	−1.52	−1.93	−1.10
8842	prominin 1	PROM1	−1.56	−1.71	−1.40
57556	semaphorin 6A	SEMA6A	−1.56	−1.55	−1.58
9687	growth regulation by estrogen in breast cancer 1	GREB1	−1.59	−1.59	−1.59
8642	dachsous 1	DCHS1	−1.60	−1.24	−1.97
650	bone morphogenetic protein 2	BMP2	−1.63	−2.29	−0.98
55303	GTPase, IMAP family member 4	GIMAP4	−1.64	−1.38	−1.89
83698	calneuron 1	CALN1	−1.80	−1.45	−2.14
147700	kinesin light chain 3	KLC3	−1.81	−1.93	−1.68
5729	prostaglandin D2 receptor	PTGDR	−2.01	−1.18	−2.85
5175	platelet endothelial cell adhesion molecule	PECAM1	−2.04	−1.54	−2.54
9619	ATP-binding cassette, sub-family G, member 1	ABCG1	−2.23	−2.50	−1.96
5121	Purkinje cell protein 4	PCP4	−2.43	−0.70	−4.17
8470	sorbin and SH3 domain containing 2	SORBS2	−2.47	−3.21	−1.73
5142	phosphodiesterase 4D, cAMP-specific	PDE4B	−2.79	−3.27	−2.31

Depicted are genes found to be significantly de-regulated in the E/R knockdown. Columns 1–3: Gene identifiers; columns 4–6: log2-fold change values for the mean of AT-2 and REH (column 4), REH (column 5), AT-2 (column 6).

### Functional annotation and pathway analysis of differentially expressed genes in the KD model

**Figure 2:**
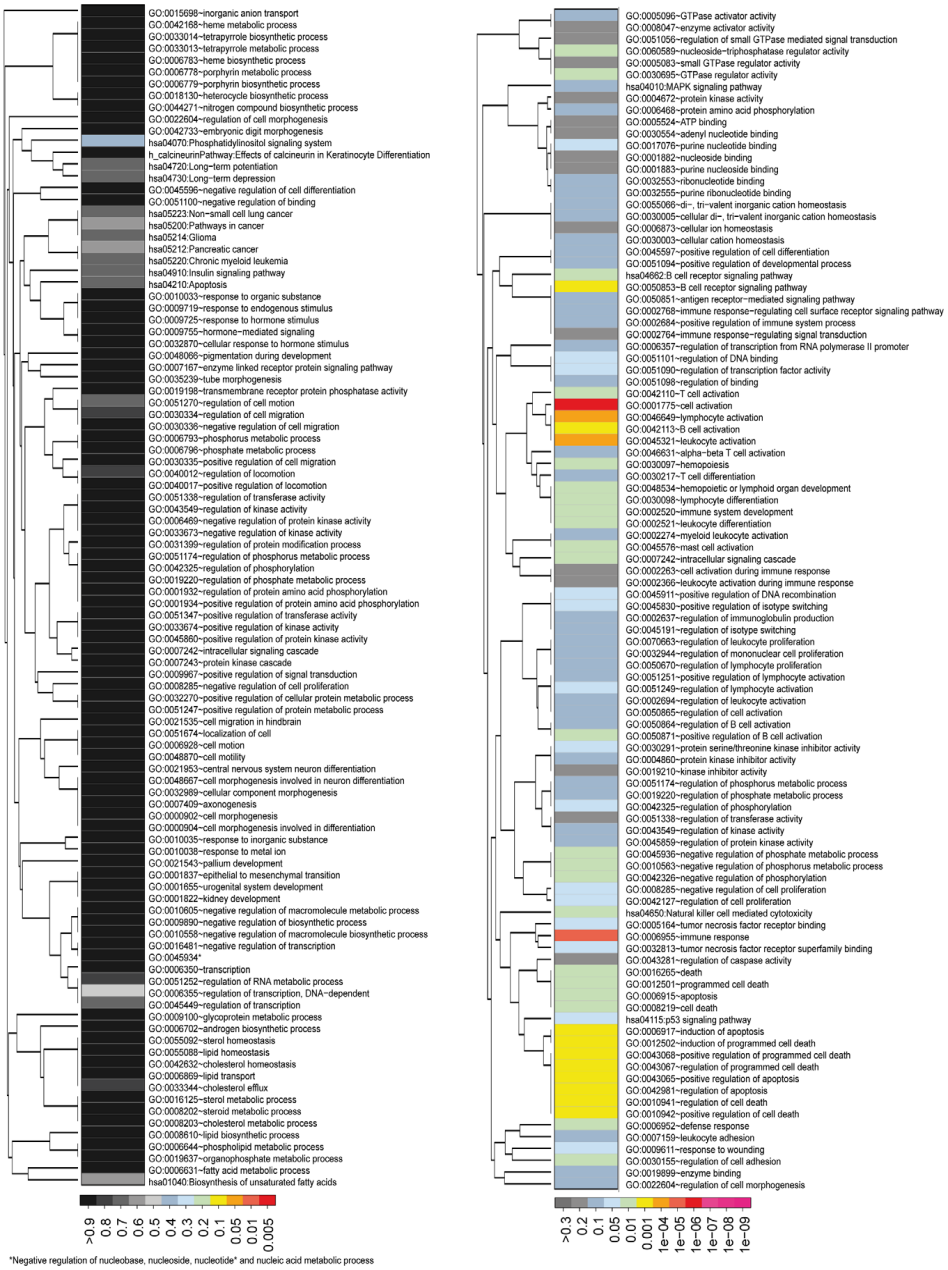
Functional annotation clustering of differentially expressed genes upon E/R KD. Visualization of the similarity of functional annotations that were determined by DAVID for down- (left) and up-regulated (right) genes upon E/R KD. The 100 most significant terms (ranked by *P*-value) are shown for both sets of annotation terms. Significance levels of functional terms are indicated by a color code shown at the bottom of the figure. Due to large differences in the range of *P*-values for the functional groups resulting from up- and down-regulated genes upon KD, a different color-scheme was used in each panel.

To systematically assess the molecular functions that are modulated by E/R, we annotated all significantly regulated genes from the E/R KD experiments according to their regulation by the fusion gene. For this purpose, we used the “Database for Annotation, Visualization and Integrated Discovery” (DAVID) [16] to classify gene lists into functionally related gene groups. The raw output from DAVID, derived from the analysis of up- and down-regulated genes (Table S2 and Table S3), was further parsed to work out more clearly the significance levels and affiliation to broader functional groups of annotation terms (Figure 2). First inspection of these functional annotations revealed a large discrepancy between E/R KD up- and down-regulated genes (Figure 2; right and left panel, respectively). While KD-UP genes significantly associate with various cellular functions and pathways, the KD-DOWN gene set, after correction for multiple testing, yielded no significant annotation term at all (the highest ranking term with *P*<0.3 was the KEGG pathway 04070:Phosphatidylinositol signaling system). These striking differences indicate that despite the similar number of up- and down-regulated genes only the KD-UP ones relate, to a high degree, to similar functions and were therefore enriched by the DAVID analysis. The KD-DOWN genes, on the other hand, do not cluster into common functions and therefore not a single term was found to be significant. Hence, the channeling of KD-UP genes to specific pathways suggests that E/R exerts its distinct and relevant gene de-regulation through repression of specific classes of target genes. Conversely, the general lack of such a KD-DOWN-related “pathway-channeling” implies that the E/R-associated up-regulation of genes might be biologically far less relevant. Alternatively, KD-DOWN genes may encode signaling pathway components that are mostly regulated by posttranslational modifications, as is, for instance, the case in the phosphoinositide-3-kinase (PI3K)/AKT/mammalian target of rapamycin (mTOR) pathway.

To test for potential direct targets of E/R, we first looked for RUNX1 consensus motifs in the promoter regions of de-regulated genes. Using gene set enrichment (GSEA) and overrepresentation analysis we could not detect an enrichment of such motifs in up- or down-regulated genes (data not shown). Second, we compiled RUNX1 targets from two very recent ChIP-seq studies [17], [18], which were derived from the analysis of human megakaryocytes and murine hematopoietic stem/progenitor cells. GSEA revealed that genes with ChIP-seq hits from both data sets are significantly up-regulated in our knockdown data. Of note, the Tijssen et al [17] data set showed a more pronounced enrichment that could be attributable to its origin from human tissue, as opposed to mouse tissue in the Wilson et al. study [18] (Table S5). Focussing on the KD-UP and KD-DOWN genes, we also found a significantly higher percentage of genes with ChIP-seq hits in KD-UP genes compared to the KD-DOWN genes (54.8% vs. 46.8%; *P* = 0.026, Fisher-Exact Test) (Table S1). These results are consistent with the notion that E/R regulates RUNX1 target genes primarily through repression [3].

**Figure 3:**
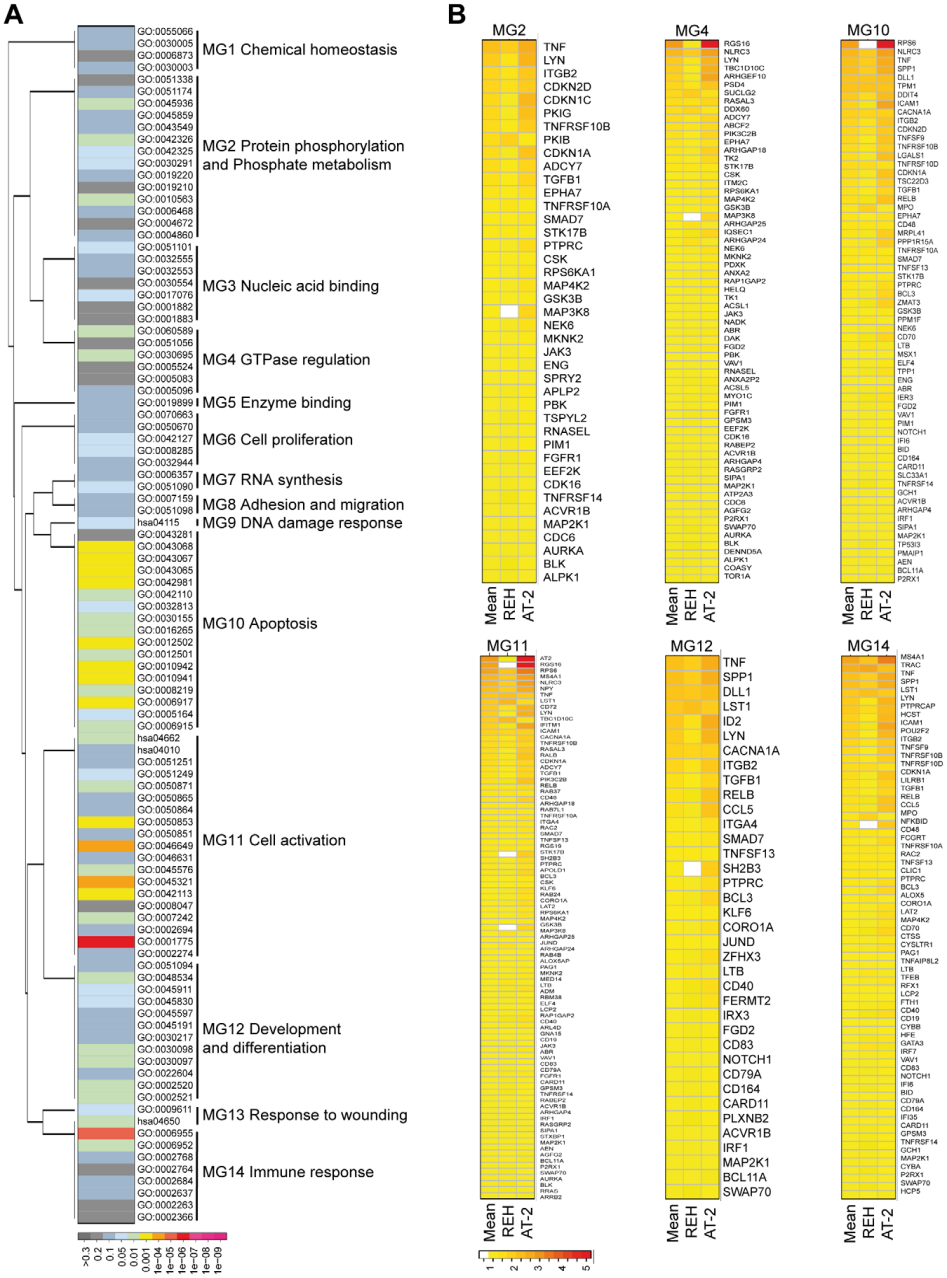
Meta-groups of functional annotations for up-regulated genes upon E/R KD. Meta-groups were curated based on gene-clustering of annotation terms. A: Top 100 annotation terms from KD-UP genes, their *P*-values and their affiliation to meta-groups. Similarity of the meta-groups was based on the number of shared genes. For distance calculations between the meta-groups genes from all contributing terms were taken together. B: Change in expression of individual genes in meta-groups that contain significant annotation terms. The color code at the bottom of the figure indicates the extent of log2-fold changes in gene expression.

Given their apparent biological relevance, we focused our further analysis on the 403 KD-UP genes and their molecular functions as well as involvement in pathways. Based on the gene-level clustering, the top 100 annotation terms were manually curated into 14 functional meta-groups (Figure 3). Note that the name of the meta-groups reflects only the most prominent annotation terms that are comprised in the respective meta-group. A list including all terms within the 14 meta-groups is shown in Table S2. Applying stringent statistical criteria (*P*<0.05), only the meta-groups “cell activation”, “immune response”, “apoptosis”, “development and differentiation”, “GTPase regulation”, and “protein phosphorylation and phosphate metabolism” were found to contain at least one significant annotation term (Figure 3A). The regulation of individual genes within the top six meta-groups upon E/R KD is shown in Figure 3B. The remaining groups (“cell proliferation”, “response to wounding”, “nucleic acid binding”, “DNA damage response”, “cell adhesion and migration”, “chemical homeostasis”, “RNA synthesis” and “enzyme binding”) contained no nominally significant annotation term.

The DAVID pathway analysis was based on the overrepresentation of “significant genes” in certain gene sets and pathways. To assess the functional impact of differentially expressed genes from the E/R KD experiments independent of a specific *P*-value threshold, we performed GSEA. This analysis resulted in many more up-regulated GO terms (147) from KD-UP than down-regulated terms (13). Importantly, these GO terms largely mapped to the same meta-groups identified in the DAVID analysis (Table S4). The same discrepancy (324 vs. 49 gene sets) held true for a large collection of >2.500 gene sets that were obtained from experimental data (“curated gene sets, C2” from MSigDB) (Table S5). The conclusions from the DAVID analysis were thus qualitatively confirmed by GSEA. Moreover, in the GSEA analysis the “Jaatinen hematopoietic stem cell UP” signature - derived from the gene expression profile of sorted cord blood CD133 (PROM1)-positive versus CD133-negative cells - emerged as the most significantly enriched set associated with the E/R KD-DOWN genes. This result may be an indicator for an intriguing new function of E/R, namely that it induces genes that are normally expressed in cord blood-derived hematopoietic stem cells [19]. To corroborate these findings, we supplemented our comparison with two other gene sets that were obtained from sorted CD34+/lineage-negative versus CD34− normal bone marrow cells designated “Andersson-UP” and “Andersson-DOWN” (microarray data were kindly provided by Andersson et al. [10]). In line with the above results, the “Andersson-UP” gene set also scored significantly in the GSEA analysis (Table S5). Combining the data from Jaatinen's and Andersson's gene sets, *CALN1*, *PROM1*, *KIT* and *CDK6* were the most highly up-regulated genes and they are similarly induced by E/R. With the exception of *CALN1*, whose function in the hematopoietic system is currently not known, all other genes are considered to be associated with hematopoietic stem or progenitor cells [20].

To the best of our knowledge, only one other group has previously analyzed the expression patterns of primary *E/R*-positive ALL cases and assigned them to GO categories [11]. Consistent with our data, they also observed a distinct association with the categories “cell differentiation”, “cell proliferation”, “apoptosis”, “cell motility” and “response to wounding”. Moreover, ectopic E/R expression in a 3T3 mouse cell line model induced the categories “adhesion” and “survival” [13].

### Genes concordantly modulated by E/R KD in leukemia model cell lines and primary ALL

Next we investigated to which extent gene expression changes that result from an E/R KD might also be reflected in a reciprocal fashion in primary ALL samples. For this purpose we used previously published data sets [8] that were generated by comparing expression profiles from *E/R*-positive with *E/R*-negative BCP ALL cases.

The ensuing “E/R ALL signature” was then compared with the E/R KD signature. Note that from the 777 significantly regulated KD genes only 409 (n = 409; 175 KD-DOWN and 234 KD-UP) were represented in the primary ALL arrays and passed initial quality filters (Table S6). Taking into account the specific regulation of these genes in primary ALL, we identified a set of genes whose expression is inversely correlated in the KD and ALL signatures. This set comprises 66 of the 175 KD-DOWN and 71 of the 234 KD-UP genes and they account for approximately one third (137/409) of the E/R signature genes present in both data sets (Table S7). In this data set, we also found a significantly higher percentage of genes with ChIP-seq hits in KD-UP genes compared to the KD-DOWN genes (67.6% vs. 48.5%; *P*<0.05 Fisher exact test) (Table S7). The top 50 regulated genes of this set are listed in Table 2. They are associated with the categories “cell activation” (*TRIB*, *FYB*, *LYN* and CD72), “immune response” (*CXCR7*, *FAIM3*, *CD48*, *CD72*, *FKBP5*), “development and differentiation” (*SPIB*, CD72, *S100A4/13*, *PLP2*), “cellular proliferation” (*LGALS1*, *CXCR7*, *SOX11*, *E2F5*, *GAB1*, *CDKN1A*, *EMP3*, *LYN*, *DDIT4*, CD72 and *LGALS1*), “cell survival” (*DRAM1*, *MDM2*, *GAB1*, I*NPP5D*, *FAIM3*, *CDKN1A*, *LGALS1*, *DDIT4*, CD72), “proliferative signaling” (*GIMAP4*, *RAC2*, *ARHGEF4*, *PSD4*), “cell adhesion and/or migration” (*DCHS1*, *CXCR7*, *PCDH, ITGA4*, *LGALS1*, *ITGB2*, *EMP3*, *S100A4*, *LYN*) and “DNA damage response” (*DRAM1*, *MDM2*, *CDKN1A*, *PSD4*). The above pathways and functions match almost perfectly with those identified in the KD model, which underscores their specific relevance for *E/R*-positive leukemia.

**Table 2:** Top 50 E/R regulated genes from microarray analysis of KD experiments and primary ALL.

Entrez gene ID	Gene name	Gene symbol	E/R KD mean	E/R+ vs. E/R− ALL
6689	Spi-B transcription factor	SPIB	2.60	−1.83
28755	T cell receptor alpha constant	TRAC	2.28	−1.03
971	CD72 molecule	CD72	1.58	−0.80
54541	DNA-damage-inducible transcript 4	DDIT4	1.56	−1.37
4067	v-yes-1 Yamaguchi sarcoma viral related oncogene homolog	LYN	1.56	−1.26
6275	S100 calcium binding protein A4	S100A4	1.45	−1.80
2014	epithelial membrane protein 3	EMP3	1.41	−0.88
3689	integrin, beta 2	ITGB2	1.31	−1.35
23550	pleckstrin and Sec7 domain containing 4	PSD4	1.30	−0.35
26112	coiled-coil domain containing 69	CCDC69	1.25	−1.50
3956	lectin, galactoside-binding, soluble, 1	LGALS1	1.11	−2.00
64777	required for meiotic nuclear division 5 homolog B	RMND5B	1.06	−0.21
1026	cyclin-dependent kinase inhibitor 1A (p21, Cip1)	CDKN1A	1.05	−0.56
51063	calcium homeostasis modulator 2	CALHM2	0.99	−1.09
9404	leupaxin	LPXN	0.95	−1.55
5355	proteolipid protein 2	PLP2	0.90	−1.47
2289	FK506 binding protein 5	FKBP5	0.89	−0.95
962	CD48 molecule	CD48	0.89	−0.81
55501	carbohydrate (chondroitin 4) sulfotransferase 12	CHST12	0.86	−0.72
9679	family with sequence similarity 53, member B	FAM53B	0.86	−1.07
10437	interferon, gamma-inducible protein 30	IFI30	0.85	−0.55
3676	integrin, alpha 4	ITGA4	0.83	−1.08
5880	ras-related C3 botulinum toxin substrate 2	RAC2	0.82	−0.53
6284	S100 calcium binding protein A13	S100A13	0.81	−0.75
272	adenosine monophosphate deaminase 3	AMPD3	0.80	−0.34
9331	beta 1,4- galactosyltransferase, polypeptide 6	B4GALT6	−0.72	1.68
10656	KH domain containing, RNA binding, signal transduction associated 3	KHDRBS3	−0.75	2.04
5101	protocadherin 9	PCDH9	−0.77	1.68
1875	E2F transcription factor 5	E2F5	−0.81	0.68
50649	Rho guanine nucleotide exchange factor 4	ARHGEF4	−0.81	3.32
9214	Fas apoptotic inhibitory molecule 3	FAIM3	−0.84	0.74
6664	SRY (sex determining region Y)-box 11	SOX11	−0.84	2.39
10402	ST3 beta-galactoside alpha-2,3-sialyltransferase 6	ST3GAL6	−0.87	0.74
26011	odz, odd Oz/ten-m homolog 4	ODZ4	−0.88	0.49
57007	chemokine (C-X-C motif) receptor 7	CXCR7	−0.91	1.19
2533	FYN binding protein	FYB	−0.91	2.48
8349	histone cluster 2, H2be	HIST2H2BE	−0.91	0.94
5095	propionyl CoA carboxylase, alpha polypeptide	PCCA	−0.94	1.38
3635	inositol polyphosphate-5-phosphatase	INPP5D	−1.03	0.37
2549	GRB2 associated binding protein 1	GAB1	−1.05	1.23
54847	SID1 transmembrane family, member 1	SIDT1	−1.06	0.92
10221	tribbles homolog 1	TRIB1	−1.09	1.11
4193	mouse double minute 2	MDM2	−1.11	0.56
55332	DNA-damage regulated autophagy modulator 1	DRAM1	−1.16	2.28
950	scavenger receptor class B, member 2	SCARB2	−1.21	0.46
57556	semaphorin 6A	SEMA6A	−1.56	1.95
8642	dachsous 1	DCHS1	−1.60	1.18
650	bone morphogenetic protein 2	BMP2	−1.63	1.53
55303	GTPase, IMAP family member 4	GIMAP4	−1.64	1.14
5142	phosphodiesterase 4D, cAMP-specific	PDE4B	−2.79	0.78

Depicted are genes found to be significantly and concordantly de-regulated by E/R in KD experiments and *E/R*-positive vs. *E/R*-negative primary BCP ALL from Ross et al. [8]. Columns 1–3: Gene identifiers; columns 4–5: log2-fold change values for the mean of AT-2 and REH (column 4) and *E/R*-positive vs. *E/R*-negative primary BCP ALL (column 5).

Two thirds (272/409) of the E/R KD signature genes that concurred with the ALL data set were not specific for *E/R*-positive ALL, but were also evident in the other subgroups. This observation evokes two, not mutually exclusive explanations, namely that these genes are either de-regulated in a similar fashion in a variety of ALL subtypes or that they represent a kind of basic but essential “BCP-ALL housekeeping gene set”. The notion that other initiating genetic events can elicit a similar gene de-regulation effect as E/R is, for instance, supported by the fact that PROM1 is also up-regulated in *MLL*-rearranged and high-hyperdiploid ALL cases, thereby counterbalancing its low expression in other ALL subtypes. Consequently, PROM1 de-regulation was not considered as being a specific feature of *E/R*-positive ALL (data not shown).

### Establishing a “malignancy signature” from the E/R KD model

E/R KD leads to profound phenotypic changes, which comprise impaired cellular proliferation, survival and leukemia reconstitution in a xenotransplant mouse model (Fuka et al., manuscript submitted). We therefore postulated the presence of a potential “malignancy signature” in the E/R KD data, whose loss would render the expression profile of treated cells again comparable to those of their normal counterpart. To test this hypothesis, we generated 10 new gene sets by comparing microarray data from primary E/R-ALL [8] with those from 5 sorted normal bone marrow derived B-cell precursor subsets [21]. Consistent with our notion, all five GSEA comparisons revealed that genes, which are up-regulated in E/R-ALL vs. normal B-cell precursors are overall down-regulated after the KD and vice versa (Table S5). Therefore, this result strongly suggests that on the gene expression level the E/R KD renders ALL cells more similar to their physiological B-cell precursor counterparts.

### Validation of selected E/R target genes by RT-qPCR

**Figure 4:**
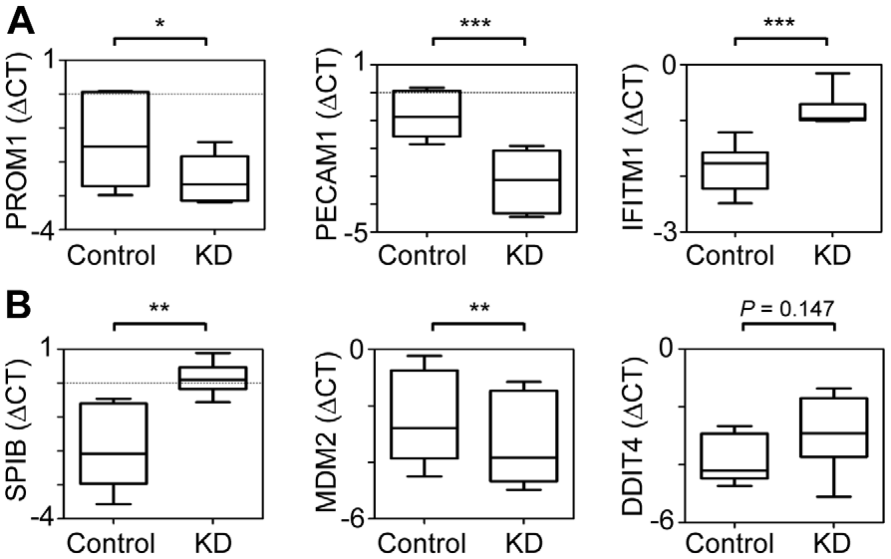
Validation of selected differentially expressed genes from the KD experiments by RT-qPCR. Quantification of transcripts of differentially expressed genes. A: Concordantly de-regulated by E/R in the KD and primary leukemias. B: Only regulated in the KD experiments. Boxes cover the median and the interquartile range (25–75th percentiles) and whiskers the minimum and maximum values. *, *P*≤0.05; **, *P*≤0.01; ***, *P*≤0.001 (paired t-test).

We validated the differential expression of several selected candidate genes contained in the KD signature, which were previously either not associated with *E/R*-positive ALL (*PROM1*, *PECAM1*, *IFITM1*; Figure 4A) or concordantly regulated in both systems (*SPIB*, *MDM2* and *DDIT4*; Figure 4B). These genes were chosen because of their potential biological relevance, since they play an important role in the context of stemness and differentiation, adhesion and migration, immune response, DNA damage response as well as apoptosis. Notably, their differential expression in the context of *E/R* is novel. Quantification results of these transcripts in both cell lines from independent KD experiments concurred with those of the microarray experiments (Figure 4).

### Functions of selected E/R regulated genes and potential implications for leukemia pathogenesis

Given that GSEA analysis of E/R KD regulated genes highlighted gene sets that are also up-regulated in hematopoietic stem cells, we chose *PROM1* (CD133) as the most prominent and attractive candidate from this set. PROM1 is implicated in maintaining stem cell properties by suppressing differentiation and has recently gained much attention as a marker of tumor-initiating cells in a variety of human cancers [22]. The fact that E/R might regulate the expression of this gene is new and intriguing and provides additional arguments to the ongoing debate dealing with the structural hierarchy of ALL and its potential replenishment from rare leukemic stem cells [23]. In favor of this notion is a recent observation, which indicates that primitive leukemia-initiating cells with long-term *in vitro* and *in vivo* proliferation capabilities are exclusively found in the CD133+CD19−CD38− cell compartment [24]. However, this observation is in contrast to the scenario proposed by le Viseur et al., which suggests that the vast majority of ALL blasts may maintain the propensity to reconstitute leukemia *in vivo* [25]. The ER-induced “stemness” expression signature, represented for instance by PROM1 and the stem cell factor ligand KIT in our model, therefore supports the later view.

The E/R-induced overexpression of stem cell markers in the respective leukemias can either be interpreted as a residual relict of a transformed primitive stem cell or, more likely, as the reflection of a continuously active stem cell program [26]. Although neither possibility excludes that the gene fusion process already occurs in a primitive hematopoietic stem cell [27], the latter requires that inappropriate stemness genes remain active or become perhaps reactivated at the level of maturation in which the bulk of the leukemic cells is arrested. This interpretation is supported by the fact that - similar to the Andersson data - *E/R*-positive leukemias cluster best with normal large pre-B II cells even after suppression of the fusion gene (data not shown) [10], [21]. It is thus tempting to speculate that up-regulation of PROM1 may play a critical role in *E/R*-positive ALL. This possibility is also relevant for our recent finding that the *E/R* fusion gene is apparently required for the *in vivo* propagation of the respective cells (Fuka et al. manuscript submitted).

*PECAM1* (CD31) encodes a homophilic adhesion receptor that mediates adhesion between endothelial cells and leukocytes and could therefore probably influence adhesion and migration of leukemic cells across the micro-vascular endothelium in various niches [28]. Since it is also contained in Andersson's CD34+ stem cell signature, we envision that its over-expression also contributes to the stem cell properties of *E/R*-positive ALL.

In contrast to the up-regulation of stem cell signature genes, genes encoding B lineage differentiation markers are frequently repressed in BCP-ALL. Thus, it is not surprising that SPIB, a B-lymphoid restricted transcription factor, is one of the genes that is strongly suppressed by E/R. Being directly induced by *paired box 5* (PAX5), the master regulator of B-lineage commitment, SPIB is a key player in B-cell development and B-cell receptor signaling [29]. This SPIB down-regulation could thus contribute to the impaired B-cell differentiation in *E/R*-positive ALL.

The E/R-associated down-regulation of IFITM1, a transcriptional target of interferon (IFN) gamma [30], fits also well into one particular point of the current concept of childhood ALL etiology, namely the one which suggests that certain forms of childhood ALL may be the unfortunate consequence of an abnormal immune response to common infections [31]. The proposed mechanism implies that inflammatory cytokines suppress the growth of normal hematopoietic cells, whereas they do not exert such an effect on, for instance, E/R-expressing cells. Consequently, fusion gene carrying cells may experience a relative growth advantage. In support of this notion it was recently shown that E/R-expressing cells are more resistant to the anti-proliferative effects of transforming growth factor (TGF) beta [32]. Since TGF beta and INF gamma are both key modulators of the immune system, one expects that the suppression of IFITM1 either concurs with or even augments these effects in response to an interferon release during common infections. In line with the proposed function of TGF beta, the suppression of IFITM1 may thus additionally fuel the expansion of an E/R-expressing leukemic clone [5].

Taking into account further mechanisms that might impair an INF gamma associated inhibition of proliferation, it is noteworthy that CDKN1A is induced via the tumor suppressor protein p53 pathway activation and leads to a G1 cell cycle arrest [33]. The attenuation of the p53 activity together with the transcriptional repression of its direct target p21, the gene product of *CDKN1A*, either by E/R-mediated repression of IFITM1 or up-regulation of the p53 inhibitor MDM2, as implied in our KD model, opens another fascinating layer of complexity to the E/R-mediated gene regulation process. Given that p53 acts as a gatekeeper of genome integrity [34], p53 down-regulation by any of the above outlined means may thus favor leukemia development. Intriguingly, MDM2 is induced by RUNX1-RUNX1T1 and may therefore be involved in the route of transformation in a similar fashion in other *RUNX1*-associated leukemias, as for instance the *E/R*-positive ones [35]. Furthermore, MDM2 may also promote tumorigenesis via a p53 independent mechanism [36]. Such findings are not only crucial for our understanding of leukemia development per se, but may be particularly helpful for the identification of especially relevant targets for tailored future therapies.

Another E/R-down-regulated gene that is involved in the p53 pathway is *DDIT4* (also known as *REDD1*). It is primarily induced by stress and negatively regulates the mTOR pathway. DDIT4 is activated by DNA damage via p53-dependent and -independent mechanisms, but also by hypoxia or energy stress [37]. Particularly this latter feature is interesting in the context of *E/R*-positive leukemia, because the majority of affected children are anemic at diagnosis, which seemingly grants hypoxic conditions a central role in their pathogenesis [38]. Noteworthy, E/R-associated DDIT4 suppression may further contribute to the observed PI3K/AKT/mTOR pathway activation and an improved cell survival [39]. Whether this suppression is a direct p53-related consequence that, as recently observed in breast cancer, also leads to hypoxia inducible factor (HIF) 1 alpha accumulation, is currently not known [40].

Taken together, the above clues reinforce the essential role that E/R plays in the entire process of leukemia development and maintenance: i) It induces genes that confer stem cell properties endowing cells with unlimited self renewal capability and simultaneously represses genes that otherwise promote differentiation; ii) it alters the DNA damage response by attenuating the p53 pathway, which in addition enables the survival and clonal expansion of cells with accumulating secondary genetic changes; iii) it triggers proliferation and cellular growth via PI3K/AKT/mTOR pathway activation, which in turn adapts extracellular signaling as well as stress and hypoxia response accordingly; iv) it also attenuates the response to inflammatory signals. All these features, sustaining proliferative signaling, evading growth suppression, resisting cell death, and induced genome instability, are typical and well established hallmarks of cancer in general [41].

Based on the analyses of our KD model, we have established a functional map of the consequences of E/R expression in an endogenous background. The modulation of various specific and more general key processes that are pivotal for leukemia pathogenesis was thus highlighted. These processes include “development and differentiation”, “apoptosis”, “adhesion and migration” as well as “DNA damage response”. Finally, these data provide also a valuable source of interesting targets and pathways whose functional validation will provide further insights into the biology of *E/R*-positive leukemia and possibly also promote the identification of novel targets for treatment.

## Materials and Methods

### Quantitative RT-PCR (RT-qPCR)

Total RNA was isolated from biological replicates of E/R-silenced REH (n = 3) and AT-2 (n = 3) cells obtained from independent KD experiments by Trizol reagent (Life Technologies, Carlsbad, CA). cDNA was synthesized by SuperScript II Reverse Transcriptase according to the manufacturer's recommendations (Invitrogen, Carlsbad, CA). Transcripts were quantified by TaqMan RT-qPCR using the ABI Prism 7900 Detection System (Applied Biosystems, Foster City, CA). The following primer/probe combinations were used: SPIB 5′-GGGCCACACTTCAGCTGTCT-3′, 5′-CAGTCCAGTCCCACAGGGAG-3′, 5′-CCTGGACAGCTGCAAGCATTCCA-3′; MDM2 5′-CACGCCACTTTTTCTCTGCT-3′, 5′-CCTGATCCAACCAATCACCT-3′, 5′-CCACCTCACAGATTCCAGCTTCGG-3′; DDIT4 5′-CTGGACAGCAGCAACAGTG-3′, 5′-CATCAGGTTGGCACACAAGT-3′, 5′-CCGGAGGAAGACACGGCTTA-3; PROM1 5′-TTGTGGCAAATCACCAGGTA-3′, 5′-TCAGATCTGTGAACGCCTTG-3′, 5′-CCCGGATCAAAAGGAGTCGGA-3′; PECAM1 5′-AGGTGTTGGTGGAAGGAGTG-3′, 5′GTGTATTGGGGCCTTTTCCT-3′, 5′-AGGCCATCCAAGGTGGGATCGT-3′ and IFITM1 5′-GGCTTCATAGCATTCGCCTA-3′, 5′-ATGAGGATGCCCAGAATCAG-3′, 5′ TCCACCGCCAAGTGCCTGAA-3′. GUSB was detected by a previously published primer/probe combination [42] and used as endogenous reference. Shown are cycle threshold Δ(CT) values.

### Gene expression analysis by microarray technology

Gene expression changes upon knockdown of E/R were followed on Affymetrix HG-U133-PLUS2 arrays (Affymetrix, Inc., Santa Clara, CA). cRNA target synthesis and GeneChip® processing were performed in the Gene Expression Profiling Unit of the Medical University Innsbruck according to standard protocols (Affymetrix, Inc., Santa Clara, CA). Microarray data were performed in compliance to MIAME guidelines and submitted to GEO - accession number GSE29639. All further analyses were performed in R statistical environment using Bioconductor packages [43].

Affymetrix CEL files were preprocessed as described previously [44], yielding a final number of 9.498 probesets that were used for all further analyses.

Differentially expressed genes were determined using a moderated t-test in the R package “limma” [45]. All *P*-values were corrected for multiple testing using the “Benjamini-Hochberg” correction method. Significantly changing genes in the E/R KD vs. control experiments were determined by calculating ratios for each gene between the two conditions for each experiment separately, thus yielding five biological replicates of relative expression for each gene (REH, n = 3; AT2, n = 2). Then, for each gene, significance was determined using a weighted one-sample t-test against the null hypothesis of no expression change (μ = 0).

For the re-analysis of primary ALL data set from Ross et al. [8], CEL files were downloaded from the St. Jude's data server and microarray data was pre-processed as described previously [44], generating a data set of 12.068 genes. In this data set *E/R*-positive vs. *E/R*-negative BCP ALL samples were compared and yielded 1.980 differentially regulated genes (*P*<0.05, moderated t-test), 1.008 of which were under- and 972 over-expressed in *E/R*-positive ALL. Combining the data sets from Ross [8] and the KD experiments a total of 5.119 genes were represented on both platforms independent of their regulation and passed initial quality filters (Table S6). This gene set was then used to look for genes that are regulated by E/R in KD experiments and primary ALL.

To test for differences in malignant vs. non-malignant cells, we analyzed *E/R*-positive ALL from the Ross data set [8] together with microarray data from five normal bone marrow B-cell precursor subsets [21] (http://franklin.et.tudelft.nl/).

### Functional annotation

The “Database for Annotation, Visualization and Integrated Discovery” (DAVID) was used to annotate the 403 up- and 374 down-regulated genes from the joint analysis of the E/R knockdown in REH and AT-2 cells. The “Functional Annotation Tool” in the online version of DAVID was run (http://david.abcc.ncifcrf.gov/) using the default parameters and focusing on the categories Gene Ontology-Molecular Function, Gene-Ontology-Biological Process and KEGG/Biocarta Pathways. All annotation terms that met the inclusion criteria were downloaded as “Functional Charts”.

### Hierarchical clustering of annotation terms

For further analysis and visualization of the similarity among annotation terms, the functional charts were first sorted by their *P*-value (corrected for multiple testing by the Benjamini-Hochberg method) and then, to determine the relationships of the top 100 annotation terms, similarity between all terms was measured by the number of their shared genes (gDist as described in Kauer et al. 2009) [44]. The matrix of pair wise gDist values (as dissimilarities: 1-gDist) for the 100 most significant terms was used as input for hierarchical clustering using the R function “hclust” in combination with the “average linkage” algorithm. Finally, the similarity among the annotation terms was visualized as dendrogram in combination with a heatmap indicating significance levels of the clustered terms. Names of meta-groups were chosen or modified from upstream gene ontology terms (http://www.geneontology.org/).

### Gene set enrichment

To define functional categories of de-regulated genes independent of a *P*-value cutoff for “significant genes”, we performed gene set enrichment analysis (GSEA) using the “pGSEA” package in the Bioconductor/R environment [46]–[48]. Gene-wise log2 expression ratios (logFC) of knockdown versus control for the cell lines REH and AT-2, and for the mean of their logFCs, were used as input for pGSEA. Gene sets were downloaded from the MSigDB v3.0 (http://www.broad.mit.edu/gsea/msigdb/ Cambridge, USA). We tested two different gene set collections available from MSigDB: curated gene sets from canonical pathways and experimental data (C2) and GO terms (C5). To validate the enrichment on genes involved in hematopoietic stem cells, we added two more gene sets to the C2 group: genes up- and down-regulated in the Andersson et al. 2005 data set (CD34+/lineage negative vs. CD34− hematopoietic cells) [10].

To test whether E/R knockdown renders the gene expression of ALL cells more similar to non-malignant cells, we added new gene sets: For each of the five comparisons of *E/R* ALL vs. normal B-cell precursor subsets we defined significantly (*P*<0.01, logFC>1.5) up- and down-regulated genes, resulting in 10 gene sets. The results for all gene sets can be found in Table S5.

To test for enrichment of putative direct RUNX1 binding targets, RUNX1 ChIP-seq data was downloaded from two sources: Tijssen et al. [17]; Wilson et al. [18].

### Deposition of microarrays

Microarray data are available online at GEO (www.ncbi.nlm.nih.gov/geo/, accession number GSE29639).

## Supporting Information

Text S1 Materials and methods (DOC)

Figure S1 **shRNA-mediated silencing of E/R leads to chimeric protein depletion.** The *E/R*-positive leukemia cell lines REH and AT-2 were transduced by lentiviral constructs encoding either the E/R specific shRNA G1 (G1) or a non-targeting shRNA (control). Protein levels of E/R (A) and RUNX1 (B) were detected by immunoblotting using anti-ETV6 and anti-RUNX1 antibodies, respectively. GAPDH was used to ensure equal loading. Numbers between bands represent the ratio between tested proteins and GAPDH quantification. A vertical line has been inserted to indicate where a gel lane was cut. These gels came from identical experiments. Shown are results from one of at least three independent E/R knockdown experiments per cell line.
(TIF)

Table S1 **Genes found to be significantly de-regulated on Affymetrix HGU-133-PLUS2 arrays from E/R KD experiments.** Columns 1–3: Gene identifiers; columns 4–6: log2-fold change values (mean of AT-2 and REH) (column 4), REH (column 5), AT-2 (column 6); column 7: *P*-value for the mean log2-fold changes of the E/R KD (from column 4) corrected by the Benjamini-Hochberg method; column 8: ChIP-seq hits from Tijssen et al. [17]; column 9: ChIP-seq hits from Wilson et al. [18].
(XLS)

Table S2 **Output from the DAVID analysis (version 6.7) for significantly up-regulated genes upon E/R KD.** The Categories “GO-Molecular Function”, “GO-Biological Process” and “Pathways” were selected for testing. Description of columns: “Category” – categories from DAVID; “Term” – specific terms within DAVID-categories; “Meta-group” – affiliation of “Term” into a meta-group, “Gene symbols” – genes from the tested gene list involved in “Term”; “Fold enrichment” – enrichment of genes involved in “Term” over random expectation; “*P*-value” – *P*-values for enrichment calculated by the EASE method used in DAVID; “Benjamini” – *P*-values corrected for multiple testing by the Benjamini Hochberg method. For details of the calculations: http://www.nature.com/nprot/journal/v4/n1/pdf/nprot.2008.211.pdf.
(XLS)

Table S3 **Output from the DAVID analysis (version 6.7) for significantly down-regulated genes upon E/R KD.** Column descriptions as for Table S2.
(XLS)

Table S4 **Results from the GSEA analysis for GO gene sets (MSigDB: C5).** Column 1: name of the MSIGDb gene set; columns 2–4: z-scores of from the pGSEA algorithm; columns 5–7: *P*-values from the pGSEA algorithm; column 8: genes involved in gene set; columns 9–12: descriptions of MSigDB gene set from MSigDB.
(XLS)

Table S5 **Results from the GSEA analysis for “curated gene sets” from MSigDB (C2).** Column descriptions as for Table S4.
(XLS)

Table S6 **All 5.119 probe sets that passed quality filters that were present on both, the HGU-133-PLUS2 arrays from our E/R KD experiments and** ***E/R*** **-positive vs.** ***E/R*** **-negative primary BCP ALL (from Ross et al.** [**8**] **; HGU-133-A arrays).** Columns 1, 2: Gene symbol (column 1) and Probe set (column 2) identifiers; columns 3, 4: log2-fold change values (column 3) and *P*-values (column 4) for the mean value of the E/R KD; columns 5, 6: log2-fold change values (column 5) and *P*-values (column 6) for *E/R*-positive vs. *E/R*-negative primary BCP ALL. Mean log2-fold change and *P*-values were calculated as described in the M&M section.
(XLS)

Table S7 **Overlap of significantly de-regulated genes from the E/R KD signature and** ***E/R*** **-positive vs.** ***E/R*** **-negative primary BCP ALL (from Ross et al.** [**8**] **).** The subset of 137 genes from Table S4 with *P*<0.05 for both, the mean from E/R knockdown vs. control and *E/R*-positive vs. *E/R*-negative primary BCP ALL. Columns 1, 2: gene identifiers; columns 3, 4: log2-fold change values (column 3) and *P*-values (column 4) for the mean value of the E/R KD; columns 5, 6: log2-fold change values (column 5) and *P*-values (column 6) for *E/R*-positive vs. *E/R*-negative primary BCP ALL; column 7: ChIP-seq hits from Tijssen et al. [17]; column 8: ChIP-seq hits from Wilson et al. [18].
(XLS)
