# Supplementary material for: The Leukemia-Specific Fusion Gene ETV6/RUNX1 Perturbs Distinct Key Biological Functions Primarily by Gene Repression
Source: PLoS One. 2011 Oct 20;6(10):e26348. doi: 10.1371/journal.pone.0026348 (PMC3197637; doi:10.1371/journal.pone.0026348)
Supplement: Text S1 Materials and methods — (DOC) [file pone.0026348.s001.doc]

**Supplementary Material and Methods**

**Cell lines**

The E/R-positive BCP leukemia cell lines REH (DSMZ, Braunschweig, Germany) and AT-2 [1] (kindly provided by J.D. Rowley, University of Chicago, IL) were both established from relapsed leukemia. They both express wt RUNX1 but have, like the majority of primary E/R-positive ALL cases, the non-rearranged *ETV6* gene deleted. Cells were cultured in RPMI 1640 medium with Glutamax (Invitrogen, Carlsbad, CA), supplemented with 10% heat-inactivated FCS, 100 IU/mL penicillin and 100 µg/mL streptomycin (all PAA Laboratories, Pasching, Austria) and kept in humidified incubators at 37° C and 5% CO_2_.

**RNA interference**

shRNA G1 (5'-CACCGGGAGAATAGCAGAATGCATCGAAATGCATTCTGCTATTC TCCC-3') is directed against the fusion region of E/R (NCBI nucleotide database accession number: S78496) and was newly designed to repress the endogenous fusion protein REH and AT-2 cells. Residues targeting RUNX1 are marked in red and those homologous to ETV6 in blue. The non-targeting shRNA LacZ (Invitrogen, Carlsbad, CA) was used as a control. Lentiviral supernatants were produced with the Block iT Lentiviral RNAi Expression System (Invitrogen), followed by quantification of lentiviral titers by ELISA-based quantification of virus associated HIV-1 p24 (Cell Biolabs, San Diego, CA) according to the manufacturer’s recommendations. Leukemia cell lines were transduced and selected for lentiviral integration with 5 µg/ml blasticidin for two weeks. Upon confirmation of E/R repression (Figure S1) mRNA was extracted between day 16-30 upon viral transduction.

**Immunoblotting**

Cell lysates were obtained with RIPA buffer supplemented with 1mM NaVO4 and 1% protease inhibitor cocktail (Roche, Basel, Switzerland). 90 µg of total proteins were resolved by 12% SDS PAGE gel electrophoresis and transferred to nitrocellulose membranes (Whatman, Kent, UK). Non-specific binding on the membranes was blocked with PBS containing 5% dry milk. Membranes were incubated with anti-ETV6 (SB89, kindly provided by P. Marynen, VIB, Leuven, Belgium), anti-RUNX1 (Active Motif, Carlsbad, CA) and anti-GAPDH (6C5, Santa Cruz Biotechnology, Santa Cruz, CA) and secondary infrared dye-labeled antibodies (LI-COR Biosciences, Lincoln, NE). Membranes were scanned with Odyssey Infrared Imaging System (LI-COR Biosciences). Intensities of bands corresponding to E/R and RUNX1 were normalized to the signal of GAPDH.

**References**

1. Fears S, Chakrabarti SR, Nucifora G, Rowley JD. (2002) Differential expression of TCL1 during pre-B-cell acute lymphoblastic leukemia progression. Cancer Genet Cytogenet 135: 110-119.
