# Supplementary figures and images for: The Leukemia-Specific Fusion Gene ETV6/RUNX1 Perturbs Distinct Key Biological Functions Primarily by Gene Repression
Source: PLoS One. 2011 Oct 20;6(10):e26348. doi: 10.1371/journal.pone.0026348 (PMC3197637; doi:10.1371/journal.pone.0026348)

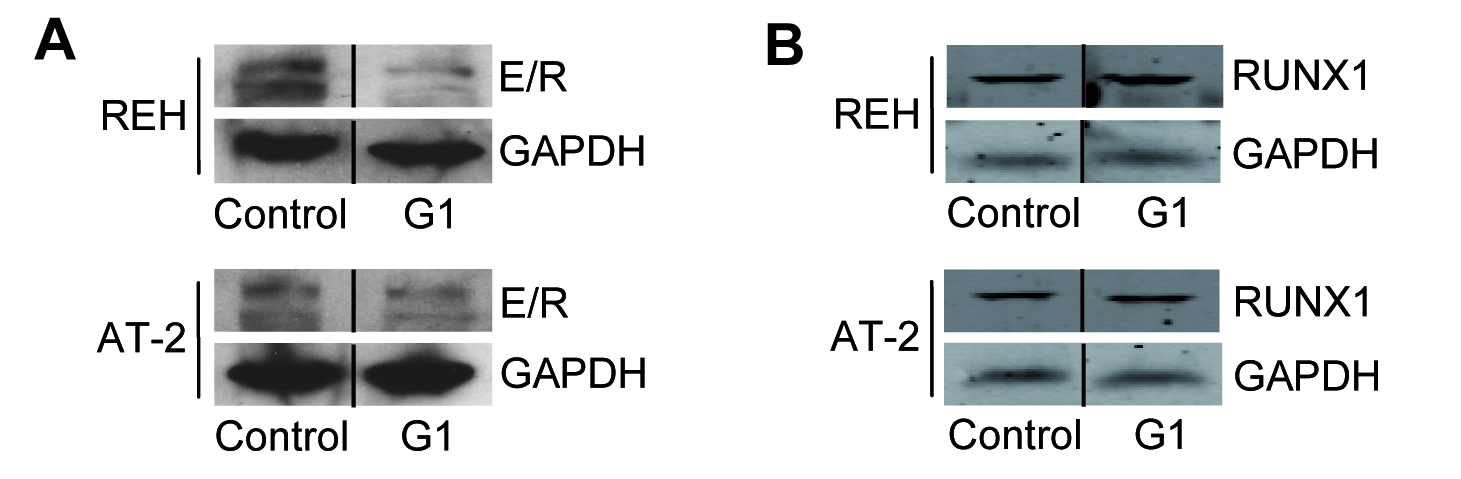

Supplement: Figure S1 — shRNA-mediated silencing of E/R leads to chimeric protein depletion. The E/R-positive leukemia cell lines REH and AT-2 were transduced by lentiviral constructs encoding either the E/R specific shRNA G1 (G1) or a non-targeting shRNA (control). Protein levels of E/R (A) and RUNX1 (B) were detected by immunoblotting using anti-ETV6 and anti-RUNX1 antibodies, respectively. GAPDH was used to ensure equal loading. Numbers between bands represent the ratio between tested proteins and GAPDH quantification. A vertical line has been inserted to indicate where a gel lane was cut. These gels came from identical experiments. Shown are results from one of at least three independent E/R knockdown experiments per cell line. (TIF) [file pone.0026348.s002.tif]
